# Development and Implementation of an Interprofessional Digital Platform to Increase Therapeutic Adherence: Protocol for a Mixed Design Study

**DOI:** 10.2196/34463

**Published:** 2022-08-12

**Authors:** Carole Anglade, Mylaine Breton, Frederic Simard, Terry Fitzpatrick, Meighen Fitzpatrick, Geneviève Bruneau, Isabelle Gaboury

**Affiliations:** 1 Department of Family Medicine and Emergency Medicine Faculty of Medicine and Health Sciences Université de Sherbrooke Longueuil, QC Canada; 2 Faculty of Medicine and Health Sciences Department of Community Health University of Sherbrooke Longueuil, QC Canada; 3 MedHelper Inc Pointe-Claire, QC Canada; 4 GMF-U de Saint-Jean-sur-Richelieu Saint-Jean-sur-Richelieu, QC Canada

**Keywords:** adherence, disease management, primary health care, electronic platform, chronic disease, design thinking, acceptability

## Abstract

**Background:**

Adherence to care plans is a major issue in health care systems. Improved adherence has several potential benefits such as ensuring treatment effectiveness and control of chronic diseases. There is currently a lack of tools to maximize treatment adherence in an integrated manner, that is, covering multiple aspects of patients’ health continuously throughout their medical care. To ensure better adherence, such tools must meet the needs of patients with chronic conditions as well as those of health care professionals. Acknowledging the health issues associated with nonadherence to treatment, an industry-research-clinical partnership aims to adapt a digital platform—facilitating patient-health care professional interactions—to improve therapeutic adherence in patients with chronic illnesses. The platform allows for exchanges between patients and health care professionals to facilitate the timing of medication use or chronic disease management and maximize patient adherence.

**Objective:**

This study aims to (1) identify the needs of patients living with a chronic condition and their health professionals concerning their interactions regarding treatment; (2) codevelop an adaptation of an interactive patient-professional platform that meets the needs identified; and (3) then test the platform and document its effects and acceptability in a clinical setting.

**Methods:**

The study will use a creative design thinking process based on the needs expressed by users (patients and health professionals) concerning treatment adherence for chronic diseases (eg, diabetes, asthma, high blood pressure, depression and anxiety, chronic obstructive pulmonary disease). A mixed method evaluation research design will be used to develop and evaluate the platform. Qualitative data will be used to assess user needs and acceptability of the platform, and quantitative data will provide the necessary insights to document its effects.

**Results:**

Technological development of the platform has been completed. Recruitment for the first part of Phase 1 started in May 2022. The results of this project to codevelop an interprofessional digital platform to increase therapeutic adherence will be relevant to clinicians and managers seeking contemporary solutions that support patient adherence to treatment for chronic diseases. These results will enable optimal use of the platform and identify areas for improvement in interactive patient-health care professional apps.

**Conclusions:**

The adoption of an interactive digital platform to facilitate effective exchanges between patients and health care professionals in primary care settings could improve adherence to treatment. The platform tested in this project takes a first step in this direction by ensuring that the technological product is developed according to the needs of patients as well as the health professionals who are likely to use it.

**International Registered Report Identifier (IRRID):**

DERR1-10.2196/34463

## Introduction

### Background

Adherence to care plans is a major issue in health care systems. According to the World Health Organization, adherence to long-term therapies for chronic illnesses in developed countries is 50%, and rates in developing countries are even lower [1]. In Canada, fewer than 51% of patients follow their prescribed treatment regimen for many chronic diseases, affecting a large portion of a population that requires strict adherence to minimize the risk of health deterioration or comorbidities [2]. Nonadherence not only leads to preventable worsening of disease [3] but also generates additional costs for the health care system and for employers due to absenteeism [4,5]. Although the responsibility for adherence has traditionally been attributed solely to patients, it is now acknowledged that some of the responsibility is shared by the professionals delivering care, with patient-professional interactions having a significant impact [6]. Therefore, adherence to medical treatment must be analyzed from a communication perspective taking into account how patients and health professionals interact and exchange health information.

Exponential growth in the number of various apps available to consumers has directly influenced the health information environment. As early as 2012, 19% of smartphone owners in the United States had previously used a health app, a usage rate that is only increasing as the population becomes more accustomed to the daily use of technology. The popularization of smartphones is changing the way users record, store, and exchange their health information and has a direct effect on patients, including improving self-management of their condition [7]. The connectivity and interactivity of health apps must be further developed to support treatment adherence in such a way that the effort required of the users to obtain or manage their health information is minimized.

Concerns have been reported about the fact that technologies are not yet designed to support data collaboration between patients and health professionals [8]. In connected health, which refers to a health care system connected with smart medical devices, communication gaps hinder progress toward higher quality health care and improvements remain to be seen [9], although projects on connected health are rapidly emerging worldwide, particularly in North America [10]. To identify needs and plan solutions, the perspective of patients has proven to be particularly relevant and valuable [11]. It is, therefore, critical to properly evaluate the user experience when developing and implementing apps to facilitate interactions between health care professionals and patients. Among the methodological approaches identified to assess users’ needs in emerging projects on connected health, design thinking has been recognized for innovative and often technological aspects that meet health care requirements [12].

### Design Thinking

Design thinking comes from the design industry and is a method designers use to “match people’s needs with what is technologically feasible and what a viable business strategy can convert into customer value and market opportunity” [13]. According to this approach, for an innovation to be successful, it should meet 3 challenges: answer the needs of users, be technically feasible, and be economically viable [14]. The design process is always confronted with the importance of the user experience: if a product does not please users, it will not be used. In this approach, a key to success is using the designer’s sensibilities; otherwise the first step—understanding the needs of future users—cannot be achieved. The designer must then rely on integrative thinking to find solutions that respond to both the needs identified and their context. During the implementation phase, potential solutions are tested using an iterative process until they are considered satisfactory. Thus, while the approach does come from the field of technological design, design thinking is characterized by a human-centered approach to problem solving [15]. It also allows for the involvement of participants with no design experience but whose expertise is necessary (such as professionals or patients) to achieve a product that corresponds to their needs through simple and engaging activities [16].

This creative process also aims to stimulate critical discussions through practical methods to bring about structural change in any type of organization [17]. In their recent review, Altman et al [18] identified 24 health care studies that used design thinking, a large majority of which were successful in achieving their objectives. Moreover, all 4 studies that compared interventions showed greater satisfaction, usability, and effectiveness using a design thinking approach than using a traditional approach [18].

### Adaptation of an Interactive Patient-Professional Platform

Acknowledging the health issues associated with nonadherence to treatment, a health technology start-up first developed an app for patient medication adherence without any patient-health care professional connectivity or interaction features. As more end users started using the app to manage their medications, the team observed that patients who used the app at the request of a health care professional (physician or pharmacist) demonstrated a higher level of engagement and adherence to their care plan. The team concluded that a web-based app designed to enable health care professionals to easily create and transfer digital care plans to patients would be an effective approach from an operational perspective for professionals and from an engagement perspective for patients. As a result, they developed a digital platform offering a patient-health care professional interaction model that seamlessly links the development, sharing, and monitoring of care plans. The platform provides postdiagnostic support to patients taking medication or following a care plan. This innovative digital health solution combines unprecedented connectivity to clinical services with a web-mobile digital platform for execution. The platform is offered as a SaaS solution and consists of 3 fully integrated components that close the loop of the entire care plan journey: (1) The web platform allows professionals or individuals to create a care plan that can consist of medications, health data measurements, and other therapies. Custom care plan activities can also be created to meet the needs of the most complex care plans. (2) Individuals can quickly activate their account and access their care plan via the mobile app. Reminders and alarms are predefined in advance by the health care professional and ready for execution by the patient. (3) Once the care plan has been accepted, patients are given access to a suite of tools to execute and report on their care plan. Some key features include push notification reminders, persistent alarms, access to medication leaflets and images, notes, a dashboard, and a report with adherence results. The report can be securely shared with a health care professional via a code so the professional can view results using the web platform.

### Objectives

This research project aims to:

- identify the needs of patients living with a chronic condition and their health professionals concerning their interactions about treatment;
- codevelop an adaptation of an interactive patient-professional platform meeting the needs identified; and
- test the platform and document its effects and acceptability in a clinical setting.

## Methods

### Study Design

The project will follow a multimethod evaluative design focused on the needs expressed by users (patients and health professionals) and is divided into 2 phases. The first phase includes a design thinking approach and consists of cyclic phases to understand user needs, identify solutions, and integrate them into a prototype. The second phase will use a pre-post design with qualitative and quantitative data analysis to document optimal use of the platform. The project will develop and test the platform in collaboration with a teaching family medicine group in Quebec that includes a broad representation of primary care practitioners.

### Procedure

**Figure 1:**
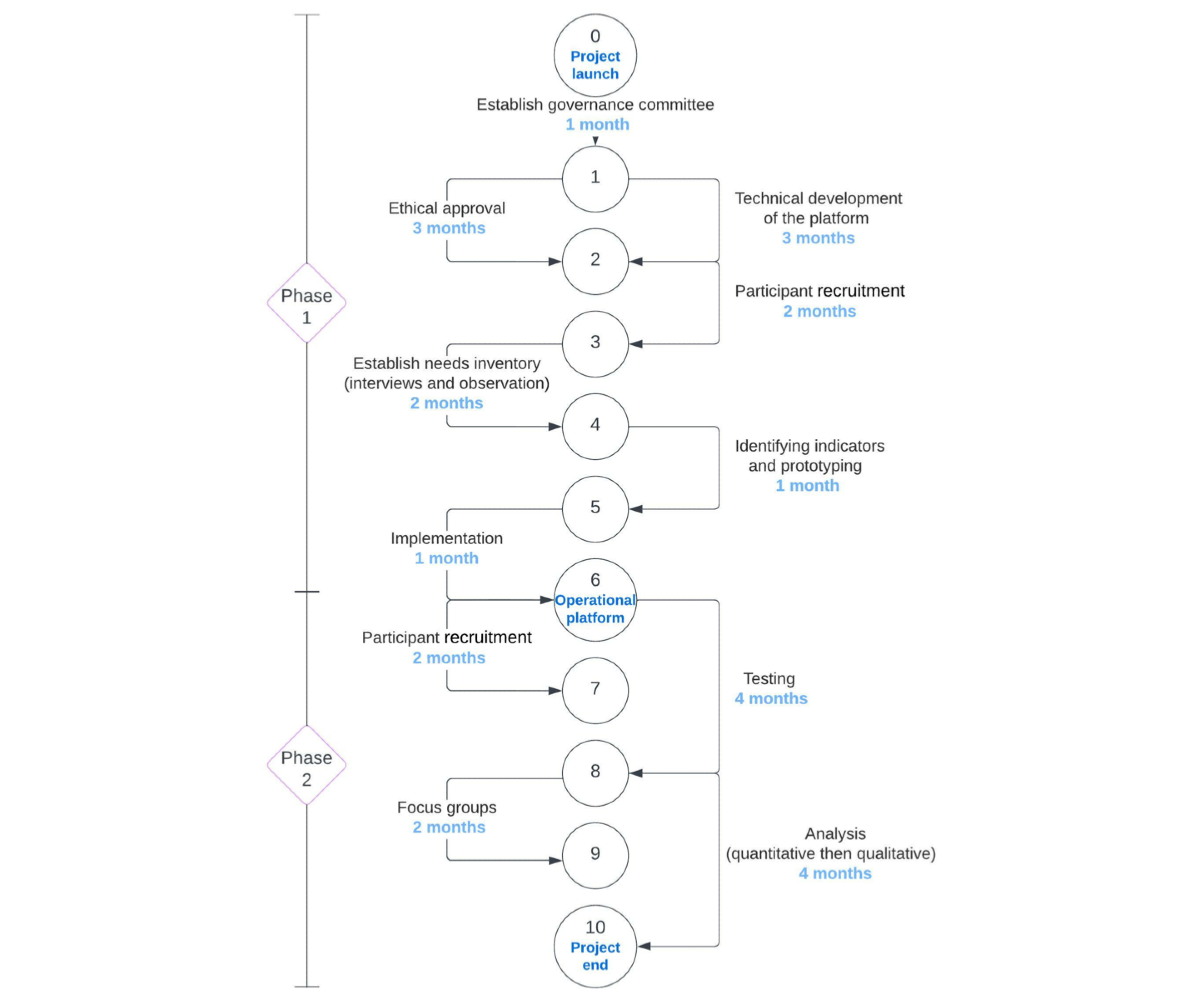
PERT diagram of the duration and tasks of the project.

Figure 1 presents the events and their dependencies of the 2 project phases.

#### Phase 1: Needs Assessment and Platform Development

##### Recruitment

The teaching family medicine group where recruitment will take place welcomes various learners: family medicine residents, medical externs, and trainees from different professions. The main missions of this clinic are providing care to the population, contributing to the training of health professionals in an interdisciplinary environment, and participating in research and teaching activities.

For this phase, we aim to recruit at least five professionals (a combination of family physicians, nurse practitioners or registered nurses, and pharmacists) and five patients from the clinic. The recruitment of health professionals will be done on a voluntary basis via email invitations that will be sent by the research team. Patients will be recruited based on a purposive sampling approach [19] guided by the recruited health professionals, who will be better able to determine the relevant pathologies to be included. Inclusion criteria for patients will include adults who speak English or French and have a chronic illness such as diabetes, chronic obstructive pulmonary disease, depression/anxiety disorders, high blood pressure, or asthma. Patients who do not have a technological tool compatible with the platform (ie, smartphone) will be excluded. Upon patient approval, the research process (including obtaining informed consent) will be explained to the patient by the research team.

##### Data Collection

To develop usable technological supports or apps, it is essential to determine the needs of users [20]. To collect the maximum number of relevant needs from participants, 2 data collection methods will be used in an effort to triangulate findings [21]. The explicit needs of patients and health care professionals will be collected through semistructured individual interviews [22]. Textbox 1 presents an excerpt of the patient interview guide.

**An excerpt of the patient interview guide.**

**Thank you for agreeing to participate in this research project. You have been invited to participate in this interview because you have one or more chronic conditions.**

What is your primary chronic illness?

1. How do you manage your (*name of disease*) on a daily basis?
2. Who are the health professionals you communicate with in relation to your (*name of disease*)?
3. What do you talk about with (*professional X*)? – *for each professional*
4. How are your exchanges with (*professional X*) going? – *for each professional*
  Who initiates the exchanges? You or (*professional X*)?
  In what way? During a visit, by phone, by email...
  How do you document the tracking of your (name of disease) to share with (*professional X*)?

**Figure 2:**
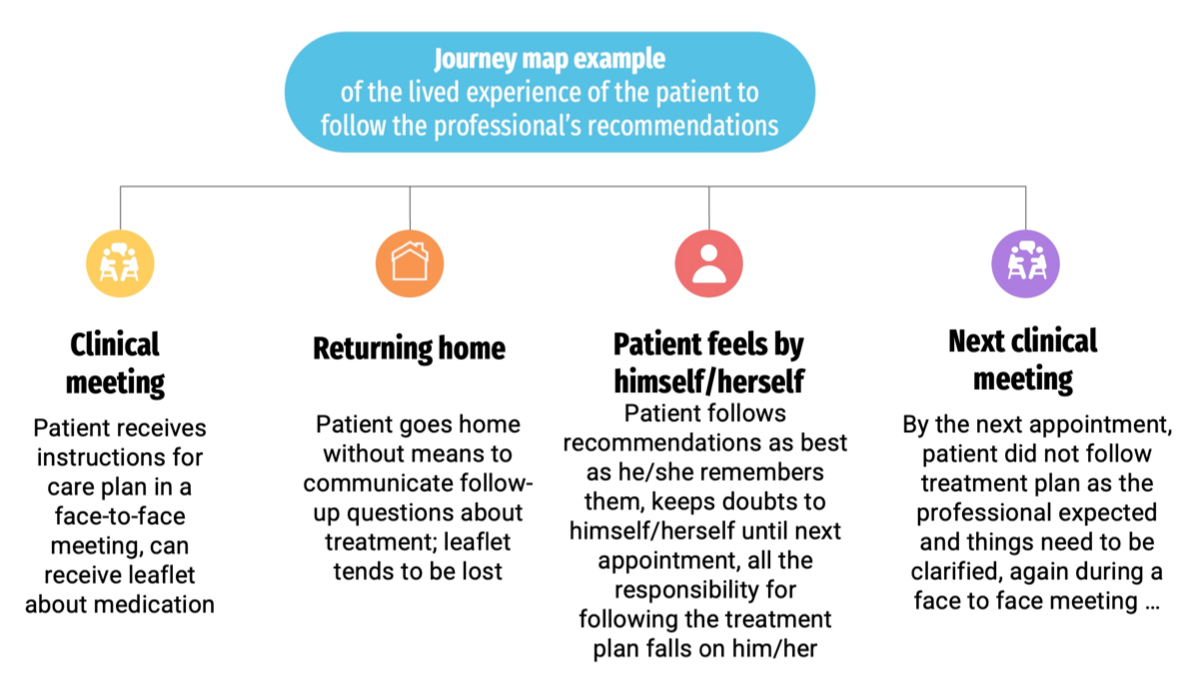
Example of a journey map.

These interviews will be used to develop a journey map of the platform’s users. Journey mapping originated in the marketing and service fields [23] and was initially used to help visualize, in detail, a particular organizational process or service, but is increasingly being used in health services [24]. They are especially useful to explore the experience of customers, users, or patients as they navigate a physical or virtual environment. In this project, the findings of the interviews will be used to draw a journey map diagram (see example in Figure 2), providing a holistic view of the patient experience.

Figure 2 presents an example of a simple journey map focused on exchanges between patients and their health professionals and the lived experience of patients as they follow the professional’s recommendations. The journey map produced in Phase 1 will include the key elements that the qualitative analysis will highlight from the data.

Next, 4-6 observations will be made of interactions between health professionals and patients during consultations for chronic disease follow-up to highlight the latent needs of these populations [20]. Latent needs are those that participants are not aware of but that can be identified by external observation of a situation. Priority will be given to interactions with different health professionals and patients with various chronic conditions. During observations, notes will be taken by a member of the research team and will include a description of the interaction between the patient and health professional, whether it takes place in person, by phone, or by text. The description will focus on the content of the exchanges as well as their modalities. These qualitative methods (interviewing and observation) will allow for observations of all aspects of the user’s objectives, needs, and preferences without relying on indirect deductions from quantitative measurements [25].

##### Analysis Strategy

Verbatim interview and observation notes will be subjected to thematic content analysis [26]. Explicit and latent needs will be used as themes for analysis. Explicit needs will be the focus of interview analyses, whereas latent needs will be the focus of observation analyses. For example, if during the observation of an interaction between a patient and physician, the patient reports that he/she did not take the blood pressure as often as the physician requested because he/she lost the paper on which the information was written, the latent need would be for the platform to offer an option for clinical recommendations to be coupled with a recall alarm. First, a member of the research team will familiarize themselves with the data by (re)reading all transcripts and notes. This will offer insights about the similarities and differences between participants’ experiences. We will then apply the following coding actions: (1) making codes, (2) organizing codes, and (3) combining patterns through an iterative process [27]. This analysis will allow for an inventory of user needs.

Both explicit and latent needs will then be translated into prototype specifications, which will be associated with metrics or indicators. For example, if a patient reports a desire to ask his/her health professional questions between clinical meetings, a prototype of the platform will be made with the specification that both the patient and the professional can initiate an exchange through the platform and that these initiations will be counted. The research team will collect these indicators for each iteration of the platform to assess the implementation of changes made through the prototyping process. Possible indicators include the following: rate of adherence to treatment as prescribed, measured as the percentage of events in the care plan that are completed by the patient versus the total number of events in the care plan; patient retention rates on the platform, which will be measured by the number of visits to the platform by patients per week; and satisfaction levels and acceptability of health care professionals and patients.

Based on the identified needs and the journey map, the development team (professionals, patients, and the research team) will reflect on the various prototypes using design thinking techniques in an iterative fashion, which implies redesigning on the basis of successive user testing [28]. During design thinking meetings (2 or 3), sketch modeling (in 2D or 3D) will be used to create a model that conveys how the participants imagine the platform. The material needed for these meetings consists, for example, of paper and pen, tape, cardboard, scissors. The research team will participate in these meetings by contributing elements based on analyses of interviews and observations. Together, the group will confirm the specifications for the prototype, which will then be implemented into the platform and discussed in an iterative manner at 1 or 2 more meetings. These iterations will focus on the user experience of professionals and patients. The technical team will be responsible for implementing the prototypes.

The development team will stop the iterative process of improving the platform, according to predefined rules [29], and proceed to Phase 2 as soon as the final prototype has been implemented as an operational version of the platform.

#### Phase 2: Pre-Post Trial of Platform Use

The second phase consists of an evaluation of the platform at the primary health care clinic. The acceptability and feasibility of the platform will be tested.

##### Recruitment

Recruitment will take place at the same clinic as in Phase 1. An email will be sent by the research team to health professionals at the participating clinic (n=23) inviting them to join the digital platform. As in Phase 1, patients will be invited by health professionals to participate in the study based on predefined criteria (eg, chronic condition, instructions required, biometric data to be collected) according to a purposeful sampling approach [19]. The same selection criteria as described in Phase 1 will be applied in Phase 2, unless the former has indicated a need to adjust these criteria. A total of 50 patients will be recruited, as well as 5-10 health professionals (physicians, resident physicians, registered and clinical nurses, pharmacists). Upon obtaining informed consent from both patients and professionals, the study steps will be explained by the research team. An automated email will then be sent by the professional to each patient via the web portal with the steps to follow to download the platform and access the information and instructions related to the care plan.

##### Data Collection

In Phase 2, the recruited patients and health professionals will use the interprofessional digital platform for 6 months. During this time, the indicators decided on in Phase 1 will be quantified by extracting data collected from the platform and the electronic medical record (EMR) of each participating patient. These data will form the quantitative database for Phase 2.

In addition, 2-3 focus groups of patient users and 1-2 focus groups of professional users will be held at the end of the testing period to provide feedback and recommendations on the platform. Users will be selected to form a convenience sample based on their level of use of the platform (low, moderate, and intensive) [30]. Recruitment for the focus groups will cease when theoretical saturation is achieved, meaning that no new codes will emerge from the analysis [31].

##### Analysis Strategy

Pre-post analyses using Student *t* tests for paired data will be performed using SPSS software (IBM, Inc.). McNemar tests will be used for dichotomous and categorical indicators.

Verbatim transcripts of group interviews will be analyzed according to a thematic content analysis [12] similar to that used in Phase 1. Quantitative data will inform on treatment adherence and patient-health care professional communication related to establishing adherence. Qualitative data will provide insights into how to promote adherence through the platform by capturing what is important to both patients and professionals when managing patients’ health conditions. This mixed methods design will allow for the data collected to form a coherent whole in order to improve the platform.

### Ethics Approval

The protocol presented in this manuscript, as well as the informed consent forms and semistructured interview guides, was reviewed and approved by the Research Ethics Committee of the Integrated Health and Social Services Centre of Montérégie-Centre (an administrative region of Quebec; study ID 2022-677) on December 7, 2021.

### Data Availability

Data sharing is not applicable to this article as no complete data sets were generated or analyzed at this stage of the study.

## Results

Technological development of the platform has been completed. Recruitment for the first part of Phase 1 started in May 2022. As shown in the Program Evaluation Review Technique (PERT) diagram, completion of both phases of the project should be achieved within 14 months, around July 2023. Study results will be available in Fall 2023. The COREQ (Consolidated Criteria for Reporting Qualitative Research) checklist [32] will be used to ensure transparent reporting of the characteristics of the findings.

## Discussion

### Anticipated Main Findings

This engaging digital platform aims to provide a new tool for health care professionals involved in managing chronic diseases. The platform will enable patient-health care professional exchanges that will facilitate chronic disease management, including the timing of medication use. During follow-ups, professionals will be able to obtain patient data in a secure manner and modify treatments or treatment plans as needed. The results of this project will be relevant to clinicians and managers seeking contemporary solutions to support patient adherence to treatment. These results will enable optimal use of the platform and identify avenues for improvement in the use of interactive patient-health care professional apps. As part of this applied research project, new functionalities will be deployed by participating health care professionals via the web portal. These new functionalities will be accessible for use in actual consultation situations via the existing web browser, distinct and separate from the operational tools currently used by professionals, such as the EMR. Efforts will be made to facilitate integration of the new functionalities into the EMR, thereby reducing required computer use and simplifying the process, once evaluation of the platform is completed and following the recommendations made in the final report. Integration of the web portal into the EMR is a critical success factor for broader rollout.

The adaptation of the platform based on the identified needs of patients living with a chronic condition and their health professionals will be acceptable to both professionals and patients.

### Comparison With Previous Work

Although the impact of mobile technologies for chronic disease management may have appeared inconsistent at first [33], as the technology was adapted and improved, it became clear that mobile health can truly support better management of chronic diseases. Studies focused on diabetes first demonstrated positive physiological and behavioral outcomes with mobile health technologies [34-37]. In 2018, a systematic review by Lee et al [38] confirmed that mobile health can also support effective behavior intervention strategies for the management of other chronic diseases, such as cancer, fibromyalgia, spina bifida, or cardiovascular diseases. One of the factors identified as enhancing self-management in patients with chronic conditions in mobile health approaches was improved communication between patients and health care providers, which is one of the key features of the platform developed in this project.

### Patient Health Benefits

It has been shown that adherence to treatment decreases rapidly and proportionally as the number of activities included in a care plan increases [39]. Unfortunately, this is a typical situation in chronic diseases, where adherence is less than 50% when the prescribed treatments include 4 or more events per day, significantly increasing the risk of patients’ health status gradually deteriorating [40]. The emergence of chronicity affects the physical and mental well-being of patients and family caregivers. By focusing on adherence, the digital platform aims to help patients and health care professionals stabilize health status and avoid the development of chronic conditions and comorbidities. Better adherence to care plans can also improve employee productivity and reduce absenteeism, an issue that has significant economic repercussions for employers [4].

### Clinical Benefits

This project will allow for experimentation with a digital platform promoting patient empowerment and the individualization of care and allow for an unprecedented level of patient-health care professional interactions as well as access to guidance and other information shared by health care professionals (medications, nonmedicated therapies, biometric data). Data collected by the patient, such as medication use, adherence rates, notes, and side effects, will be accessible to health professionals via the web portal. This research project aims to demonstrate that the higher level of patient empowerment expected or to be achieved through this interactive and integrated (web and mobile) digital platform will improve adherence to prescribed care plans.

### Benefits for Health Care Professionals

Information quality gains are expected for health care professionals when users of the digital platform return to the clinic. Professionals will be able to access a web portal interface specifically designed to view patient care and biometric data in real time using a secure code generated by the patient via the digital platform. This new approach will allow physicians and nurses to view medications, other care plan elements, biometric measures, and patient adherence rates in less than a minute via a standardized web interface. Direct productivity gains are also expected for professionals relative to the traditional approach, which is based on verbal exchanges with patients.

### Operational Benefits

The study will also test new ways of performing traditionally manual tasks that can now be done via the new platform, such as distributing notes and patient instructions. This new way of communicating treatment-related information will not only allow the patient to obtain instructions effortlessly via the digital platform, but also, as a bonus, eliminate the risk of instructions being misplaced. Therefore, this project plans to observe the impact of the platform on patient follow-up on information provided and potential lightening of the task load of health professionals, a factor that may promote their adoption of the platform. In addition, the project will allow us to observe, from an intrinsic perspective, impacts for professionals, a nonnegligible notion in a context that is sometimes difficult for human resources (particularly during the pandemic, when patient-health care professional interactions were constantly adapting).

### Strengths and Limitations

As shown by Ahmed et al [41], there are now multiple technological options aimed at improving adherence to treatment, at least medication adherence. However, the lack of studies that included health care professionals during app development is concerning. In their review, only 57 of the 420 free apps identified were developed with the involvement of health care professionals. One of the strengths of our study is the inclusion of both patients and health professionals, from exploration of their needs at the beginning, to their active participation during elaboration of the prototype, to testing of the platform in their practices. Furthermore, the efforts that will be made to allow communication between the platform and the EMR will be a significant benefit over other existing apps. As a feasibility research study, this project does not involve a control group nor does it have an effective sample size. However, our aim is not to demonstrate a generalizable effect of the platform on the management of chronic diseases but intends to create a tailored and relevant tool to be implemented in an authentic clinical setting.

### Conclusions

This research project will explore in-depth the use of an adapted interprofessional digital platform to maximize treatment adherence in an authentic clinical environment. By creating a model of patient-primary health care professional interactions, this platform will ease the process of developing, sharing, and monitoring care. The adoption of an interactive digital platform to facilitate effective exchanges between patients and health care professionals in primary care settings could improve adherence to treatment. The platform tested in this project takes a first step in this direction by ensuring that the technological product is developed according to the needs of patients as well as the health professionals who are likely to use it.

## Appendix

Multimedia Appendix 1 Results of the 4th Call for projects of the Fonds de Soutien à l'innovation en Santé et Services Services (FSISSS).

## Abbreviations

COREQ: Consolidated Criteria for Reporting Qualitative Research
EMR: electronic medical record
PERT: Program Evaluation Review Technique
