# Supplementary material for: Development and Implementation of an Interprofessional Digital Platform to Increase Therapeutic Adherence: Protocol for a Mixed Design Study
Source: JMIR Res Protoc. 2022 Aug 12;11(8):e34463. doi: 10.2196/34463 (PMC9419043; doi:10.2196/34463)
Supplement: Multimedia Appendix 1 [file resprot_v11i8e34463_app1.pdf]

Montréal, le **14 avril 2021**

À l'intention des responsables de projet et de leurs collaborateurs.

Objet : Résultats du 4e Appel à projets du Fonds de Soutien à l'innovation en Santé et Services sociaux (FSISSS) — Projet **4-1-17** intitulé « **Implantation d'une plateforme numérique visant à accroître l'observance thérapeutique via un modèle interactif patient-professionnel de la santé** » de **MedHelper Inc.** avec le **Centre intégré de santé et services sociaux de la Montérégie-Centre**.

Mesdames/Messieurs,

Nous vous remercions de l'intérêt que vous et votre équipe avez démontré de prendre part à l'intégration d'innovations dans le réseau de la santé et des services sociaux. Nous sommes conscients des efforts que vous et votre équipe avez dû fournir afin de soumettre votre projet dans des échéanciers serrés au deuxième dépôt du 4e Appel à projets du Fonds de soutien à l'innovation en santé et services sociaux (FSISSS), lancé le 26 octobre 2020.

Le processus de sélection inclut une rétroaction des autorités du ministère de la Santé et des Services sociaux (MSSS). Cette rétroaction permet à l'ensemble des équipes de recevoir les commentaires et suggestions du MSSS quant à la pertinence et la faisabilité de leur projet. L'avantage de cette démarche est de permettre un meilleur arrimage avec les orientations ministérielles et de structurer les projets afin d'augmenter leur chance d'intégration.

À cette fin, un Comité de pertinence a été mis en place par le Bureau de l'Innovation. Ce comité, composé de sous-ministres adjoints du MSSS, a analysé l'ensemble des projets admissibles.

À la suite de cette étape, les projets retenus par le Comité de pertinence sont présentés au Comité d'évaluation, formé de groupes d'experts reconnus dans leur domaine. Ces évaluateurs ont été rigoureusement sélectionnés sur la base de leurs expériences professionnelles et de leurs compétences pertinentes, en lien avec chacun des projets qui leur sont confiés.

Nous avons le plaisir de vous annoncer que la demande susmentionnée a été retenue. Vous recevrez sous peu une entente de financement à signer.

Attention : Nous vous demandons d'attendre que l'annonce officielle des résultats du 4e Appel du FSISSS soit faite avant de partager publiquement cette nouvelle.

740, rue Notre-Dame Ouest
Bureau 1400, Montréal (Québec), H3C 3X6
T : +1 (514) 398 0896

www.medteq.ca
info@medteq.ca

À titre d'information, voici les commentaires reçus suite à la revue de votre plan d'affaires unique (PAU) par les deux comités.

Commentaires du Comité de pertinence :

Le Comité a jugé que le projet était pertinent. Il conseille cependant de clarifier les retombées du projet.

Commentaires du Comité d'Évaluation :

Forces :

- Le projet présente une excellente diversité et qualité des intervenants : médecins sur le terrain, chercheurs de renom, gestionnaires expérimentés et supportés par deux plus petites entreprises MedHelper et Nexapp Inc. et une entreprise nationale (TELUS). L'entente avec TELUS post-projet ajoute beaucoup de confiance.
- Le Comité d'évaluation a jugé intéressant de cibler les infirmières et les IPS.
- L'implication des patients dans le projet et son développement représente également une force et une valeur importantes.
- La stratégie de déploiement ainsi que le plan de gestion de changement sont très réalistes et reflètent la situation actuelle sur le terrain.

Faiblesses :

Caractère innovant et compétition :

- Les évaluateurs ont manqué d'information sur le contexte actuel et le paysage compétitif pour être en mesure de bien évaluer la portée du projet et le caractère innovant de la solution ?

Méthodologie :

- La mesure du succès a suscité des interrogations au sein du Comité. Une grosse partie du projet est pour le UX qui permettra d'adapter le logiciel au nouveau contexte, mais pas à l'acquisition de données. Dans le guide, on mentionne que les activités permettant d'améliorer l'efficacité intrinsèque de la solution sont exclues. Est-ce que le UX fait partie de cette catégorie ?
- Après combien de temps l'adhésion d'un patient à son plan de soin est-elle considérée comme un succès ?
- Si l'on allonge la période d'adhésion moyenne de deux semaines, est-ce un succès ou un échec ?

740, rue Notre-Dame Ouest
Bureau 1400, Montréal (Québec), H3C 3X6
T : +1 (514) 398 0896

www.medteq.ca
info@medteq.ca

- Le taux d'adhésion est autorapporté. Il est important que le taux d'adhésion initial soit mesuré. Le questionnaire aux usagers est donc important et il est nécessaire de savoir de quel type d'usager il est question (niveau de littératie, etc.).

Pistes d'améliorations et autres commentaires :

- Une certaine partie de la population n'est pas suffisamment à l'aise avec l'utilisation de ressources internet et électronique, ce qui réduit leurs capacités à utiliser des outils de santé numérique. Cette partie de la population peut également être atteinte de maladies chroniques. Une approche complémentaire pour les atteindre devrait aussi être considérée.
- Il manquait des informations concernant la gestion, le partage et la sécurité des données des patients. Le Comité d'évaluation s'est demandé si les données seraient uniquement utilisées pour développer le projet et l'application ou si elles seraient par la suite revendues.
- La structure de coût concernant le coût récurrent mensuel de 100 $ par professionnel de la santé pour la participation sur 24 mois (page 8/37) pendant le projet de recherche n'est toujours pas claire. Ceci suscite également un questionnement pour le futur déploiement de l'application qui pourrait être compromis par un coût mensuel trop élevé pour les médecins (est-ce qu'un coût de 1200 $ démotivera certains médecins ? Le coût des licences de DME varie entre 2000 et 2500 $ par années, partiellement subventionnées).
- La faiblesse notée par le comité de pertinence concernant l'intégration avec les DME a été prise en compte par le responsable du projet qui a obtenu une lettre d'engagement de Telus qui s'engage à r collaber pour un arrimage avec leur DME en place, ce qui très positif pour le Comité.
- Le Comité d'évaluation jugerait intéressant d'étendre la portée du projet à d'autres maladies chroniques ou à de la prévention (ex : dyslipidémie, arthrose, activité physique, etc.) et d'étendre l'accent sur les interactions ou délégations interdisciplinaires de première ligne (ex. loi permettant actes pour pharmaciens).
- La lettre d'intention de Telus, tout particulièrement l'entente contenant la condition de l'approbation nécessaire au Bureau de certification et homologation (BCH), peut impliquer un processus complexe et onéreux de la part des partenaires. Ceci n'est pas inclus dans le cadre du projet, mais si ce n'est pas déjà fait, il serait judicieux de l'estimer.
- Le modèle d'affaire d'association aux entreprises de dossiers médicaux électroniques est à privilégier et primordial pour avoir une plus-value et une certitude de diffusion de l'application de Medhelper. Ceci générerait plus de certitude quant à l'atteinte d'un nombre de patients maximisé. L'application Medhelper devrait être un module du portail patient avancé de plus en plus offert par les DME. De ce fait, les associations avec Telus et Medesync sont donc importantes, mais ne devraient pas seulement être exclusives à

740, rue Notre-Dame Ouest
Bureau 1400, Montréal (Québec), H3C 3X6
T : +1 (514) 398 0896

www.medteq.ca
info@medteq.ca

Telus. L'application bénéficierait d'être également offerte aux autres DME accrédités par le bureau d'homologation (ceci est bien mentionné à la page 16 de la demande).

- Le Comité d'évaluation a également soulevé certaines questions :
  - Bien que le temps investi par le personnel soignant générera probablement des économies de temps futures, qu'en est-il de la réalité actuelle ? Quelles activités devraient potentiellement être sacrifiées au profit de l'investissement de temps requis par les professionnels de la santé ?
  - Pourquoi n'y a-t-il aucune implication du pharmacien ? Il s'agit d'un intermédiaire important qui peut jouer un rôle dans l'adhérence surtout avec les nouveaux modèles de pharmacie.
  - La gestion du changement est importante pour le professionnel et les usagers. Comment cela s'imbrique-t-il dans leur quotidien ? Quel lien est fait avec les ententes télésanté (et l'engouement) en cours avec les médecins de famille ?

MEDTEQ+ et le Bureau de l'innovation sont heureux de soutenir ce projet, qui a le potentiel de fournir des solutions innovantes aux défis auxquels fait face le réseau de la santé du Québec.

Veuillez agréer, Mesdames/Messieurs, l'expression de nos sentiments les meilleurs.

Diane Côté

Présidente-directrice générale de MEDTEQ+

740, rue Notre-Dame Ouest
Bureau 1400, Montréal (Québec), H3C 3X6
T : +1 (514) 398 0896

www.medteq.ca
info@medteq.ca
